# Dense121GAN: transfer learning-enhanced conditional generative adversarial network with DenseNet121 for reliable and efficient segmentation in medical and industrial imaging

**DOI:** 10.7717/peerj-cs.4020

**Published:** 2026-07-07

**Authors:** Muhammed Davud

**Affiliations:** Department of Software Engineering, Istinye University, Istanbul, Turkey

**Keywords:** GAN-based segmentation, Dense121GAN, Image segmentation, Transfer learning, cGAN

## Abstract

Accurate image segmentation in medical and industrial domains remains challenging due to small object sizes, complex textures, and diverse defect morphologies. To address these limitations, we propose Dense121GAN, a conditional generative adversarial network (cGAN) that integrates a pre-trained DenseNet121 as a frozen encoder. The proposed architecture promotes effective feature reuse and stable information flow through residual skip connections. By leveraging transfer learning, Dense121GAN captures rich hierarchical representations and improves training stability, particularly in data-constrained settings. Extensive experiments were conducted using five-fold cross-validation across 16 heterogeneous datasets. Dense121GAN consistently outperformed UNet and ResNet-based generative adversarial networks (GANs) across multiple evaluation metrics, including Intersection over Union (IoU), Dice coefficient (Dice), Matthews Correlation Coefficient (MCC), Cohen’s Kappa, Structural Similarity Index Measure (SSIM), coefficient of determination (*R*^2^), and Symmetric Mean Absolute Percentage Error (SMAPE). The model also demonstrated stable training behavior and mitigated common GAN failure modes such as mode collapse. In addition, Dense121GAN achieved faster convergence compared to the evaluated baseline architectures. These results highlight the effectiveness, robustness, and computational efficiency of Dense121GAN. The proposed framework provides a practical and versatile solution for both clinical diagnostic and industrial inspection applications.

## Introduction

Image segmentation is a fundamental task in computer vision with important applications in both industrial and medical domains (Shahzad et al., 2024; Hector & Panjanathan, 2024). In industrial settings, it supports defect detection, robotic inspection, and quality control (Sime et al., 2024), while in medical imaging it enables accurate delineation of anatomical structures and pathological regions for diagnosis and treatment planning (Wang et al., 2022). Traditional segmentation approaches, including thresholding, edge detection, region-based methods, and clustering algorithms, are computationally efficient but often struggle with noise, low contrast, heterogeneous textures, and multi-scale structures commonly encountered in real-world images (Abdelsamea, 2015; Xu et al., 2024; Zhang et al., 2010).

The emergence of deep learning, particularly convolutional neural networks (CNNs), significantly improved segmentation performance. Architectures such as U-Net (Ronneberger, Fischer & Brox, 2015) and ResNet (He et al., 2016) enhanced feature learning through encoder-decoder structures, skip connections, and residual learning. These models achieved strong performance across various image synthesis and segmentation tasks (Chartsias et al., 2017; Zhu et al., 2017; Zhang et al., 2019; Hoffman et al., 2018). However, CNN-based architectures remain dependent on large annotated datasets and often struggle to capture long-range contextual dependencies.

To further improve image generation and segmentation, researchers introduced Generative Adversarial Networks (GANs) (Goodfellow et al., 2014). Conditional GANs (cGANs), such as pix2pix (Mirza & Osindero, 2014), extended adversarial learning to image-to-image translation tasks and have been widely applied in medical image synthesis and industrial defect simulation (Frid-Adar et al., 2018; Kumar et al., 2024; Hayajneh et al., 2025). Nevertheless, GAN-based architectures still face challenges including training instability, checkerboard artifacts, mode collapse, and slow convergence (Odena, Dumoulin & Olah, 2016; Naeem et al., 2025; Chakraborty et al., 2024). These limitations continue to motivate the development of more robust and efficient segmentation frameworks.

Transfer learning has become a widely adopted strategy for improving performance in limited-data settings by leveraging pretrained representations from large-scale datasets such as ImageNet (Gupta, Pathak & Kumar, 2022). Among existing architectures, DenseNet121 (Huang et al., 2017) has demonstrated strong discriminative performance due to its dense connectivity, efficient feature reuse, and improved gradient propagation (Spanhol et al., 2015; Shen et al., 2015). Although DenseNet121 has previously been used as a feature extractor or discriminator backbone in GAN-based frameworks (Davud, 2024), its integration as the encoder within a cGAN generator for segmentation tasks remains largely unexplored.

To address this gap, this article proposes Dense121GAN, a pix2pix-based conditional GAN that integrates a pretrained DenseNet121 encoder within the generator architecture. The proposed framework leverages transfer learning and residual skip connections to improve feature reuse, gradient propagation, segmentation stability, and convergence behavior, particularly in heterogeneous and data-constrained scenarios.

### Contributions of this work

This article introduces Dense121GAN, a conditional GAN (cGAN) framework that integrates DenseNet121 within the generator architecture to improve segmentation accuracy, feature reuse, and training stability. The main contributions are summarized as follows:

1. **Integration of DenseNet121 into the generator:** A pretrained DenseNet121 encoder is integrated within the cGAN generator to improve feature reuse, gradient propagation, and segmentation performance, particularly in limited-data settings.
2. **Residual skip connections for feature preservation:** Four residual skip connections are introduced between the encoder and decoder to preserve spatial and structural information during reconstruction.
3. **Improved robustness and training stability:** Dense121GAN mitigates common GAN failure modes, including training instability and mode collapse, through stabilized information flow and efficient feature reuse.
4. **Enhanced training efficiency:** The proposed architecture achieves faster and more stable convergence during training, improving optimization efficiency and reducing computational cost.
5. **Strong comparative performance:** Dense121GAN consistently outperforms the evaluated UNet and ResNet-based GAN models across most datasets while maintaining competitive performance against reported state-of-the-art methods.

### Paper organization

The remainder of this article is organized as follows. ‘Related Work’ reviews related studies on GAN-based segmentation and DenseNet architectures. ‘DenseNet121’ describes the DenseNet121 architecture. ‘Materials and Methods’ presents the proposed Dense121GAN framework, including the generator, discriminator, loss functions, datasets, preprocessing pipeline, and evaluation protocol. ‘Results’ presents the experimental results across medical and industrial datasets, while ‘Discussion’ discusses the obtained findings and analyzes the robustness and behavior of the proposed framework. Finally, ‘Conclusion’ summarizes the main conclusions and outlines future research directions.

## Related work

Image segmentation has evolved from traditional rule-based techniques to deep learning-driven approaches, particularly in medical and industrial applications. Classical methods such as thresholding, edge detection, and region-growing are computationally efficient but often struggle with noise, low-contrast regions, and structurally complex images (Depto et al., 2021). To address these limitations, convolutional neural networks (CNNs), especially U-Net and its variants, became dominant due to their encoder-decoder structures and skip connections, which enable effective feature extraction and precise localization.

Several studies have enhanced U-Net using preprocessing, residual learning, and attention mechanisms. In leukocyte segmentation, adaptive Retinex preprocessing combined with U-Net improved robustness under uneven illumination (Chen & Zhu, 2022). CellSegUNet incorporated residual and attention modules to improve feature representation and reduce semantic gaps (Metlek, 2024). Comparative evaluations on blood cell segmentation further demonstrated strong performance of DeepLabv3+ and U-Net-based architectures (Toptaş & Hanbay, 2023). Similarly, DenseNet-, ResNet-, and EfficientNet-based encoders combined with attention modules such as Convolutional Block Attention Module (CBAM) improved breast tumor segmentation performance by refining spatial and channel-wise feature representations (Anari et al., 2025).

In chest X-ray segmentation, several approaches integrated reconstruction, attention, and residual learning strategies. Reconstruction-based CNN frameworks improved segmentation of opacity-affected lung regions (Souza et al., 2019), while TB-UNet incorporated dilated fusion and attention modules to enhance multi-scale feature extraction (Iqbal, Usman & Ahmed, 2023). Attention ResU-Net and related variants further improved gradient propagation and localization performance through residual and attention mechanisms (Jena et al., 2024). Additional studies demonstrated the effectiveness of deeper U-Net architectures and joint segmentation-classification frameworks for improving chest X-ray analysis and interpretability (Sharma et al., 2022; Kamil & Hashem, 2022; Rashid, Akram & Hassan, 2018).

Polyp segmentation has also seen substantial advances through attention-based and transformer-enhanced architectures. EfficientNetB0 combined with Squeeze-and-Excitation (SE) blocks improved lightweight segmentation performance (Erol & Sarıkaya, 2022), while Squeeze and Multi-Context Attention (SMCA) enhanced cross-dataset generalization (Bhattacharya et al., 2023). Transformer-enhanced architectures such as FCBFormer with SwinV2 achieved strong segmentation accuracy by improving global contextual modeling (Fitzgerald & Matuszewski, 2023). Other approaches incorporated ensemble learning, alternative color spaces, and selective feature aggregation with boundary-aware losses to improve robustness and boundary delineation (Aghalari & Bizaki, 2024; Fang et al., 2019).

UNet++ further improved multi-scale feature aggregation through nested skip pathways and deep supervision, outperforming classical U-Net across multiple datasets (Zhou et al., 2019). In industrial inspection, anomaly segmentation methods increasingly rely on autoencoders, transformers, and adaptive inference strategies. CNN autoencoders with SSIM-based reconstruction loss demonstrated effective anomaly localization (Napoletano, Piccoli & Schettini, 2021), while transformer-based frameworks such as MMCF improved subtle defect detection through multi-scale masking and attention-enhanced feature fusion (Liu et al., 2024). Other approaches, including Test-Time Training and support vector data description (SVDD)-based embedding methods, enhanced robustness and generalization in industrial anomaly segmentation tasks (Costanzino et al., 2024; Tsai, Wu & Lai, 2022).

Recent research has also explored state-space models, transformer-integrated GAN architectures, foundation-model-based segmentation, and multimodal segmentation frameworks. Architectures such as SegMamba and Serp-Mamba enable efficient long-range dependency modeling, particularly for volumetric and curvilinear medical structures (Xing et al., 2024; Wang et al., 2024a). In parallel, SegDINO leverages self-supervised pretrained vision backbones to provide semantically rich representations with lightweight decoders (Yang et al., 2025). Transformer-integrated GAN architectures such as T-GAN have also been proposed to improve global contextual modeling and long-range dependency representation in adversarial segmentation frameworks through transformer-style attention mechanisms (Reddy et al., 2026).

Recent multimodal segmentation approaches further incorporate language guidance, temporal reasoning, and multimodal transformers to improve segmentation performance in complex scenarios. Examples include referring video instrument segmentation, end-to-end referring video object segmentation with multimodal transformers, and language-driven interactive segmentation frameworks (Wang et al., 2024c; Botach, Zheltonozhskii & Baskin, 2022; Wang et al., 2024b). These approaches can provide stronger contextual understanding when additional modalities such as textual or temporal information are available. However, they often require more complex annotations, multimodal supervision, and substantially larger training pipelines.

In contrast, Dense121GAN focuses on reliable image-only segmentation under heterogeneous and limited-data settings through transfer learning, dense feature reuse, residual skip connections, and adversarial optimization.

Overall, recent segmentation research demonstrates a consistent trend toward integrating advanced encoders, attention mechanisms, multiscale feature learning, pretrained representations, and efficient optimization strategies to improve robustness and segmentation accuracy across diverse medical and industrial imaging tasks.

## Densenet121

DenseNet121 (Huang et al., 2017) is a deep convolutional neural network characterized by dense connectivity, where each layer receives feature maps from all preceding layers. This design improves feature reuse, enhances gradient propagation, and reduces parameter redundancy. The architecture consists of 121 layers with convolution, batch normalization, and ReLU operations, using a growth rate of 32 feature maps per layer.

As illustrated in Fig. 1, DenseNet121 is organized into four dense blocks separated by transition layers that reduce spatial dimensions while preserving feature depth. The network begins with a 7 × 7 convolution and max-pooling layer, followed by dense blocks containing 6, 12, 24, and 16 layers, respectively. Transition layers employ 1 × 1 convolutions and average pooling for feature compression and downsampling. The architecture concludes with global average pooling and a fully connected classification layer.

**Figure 1:**
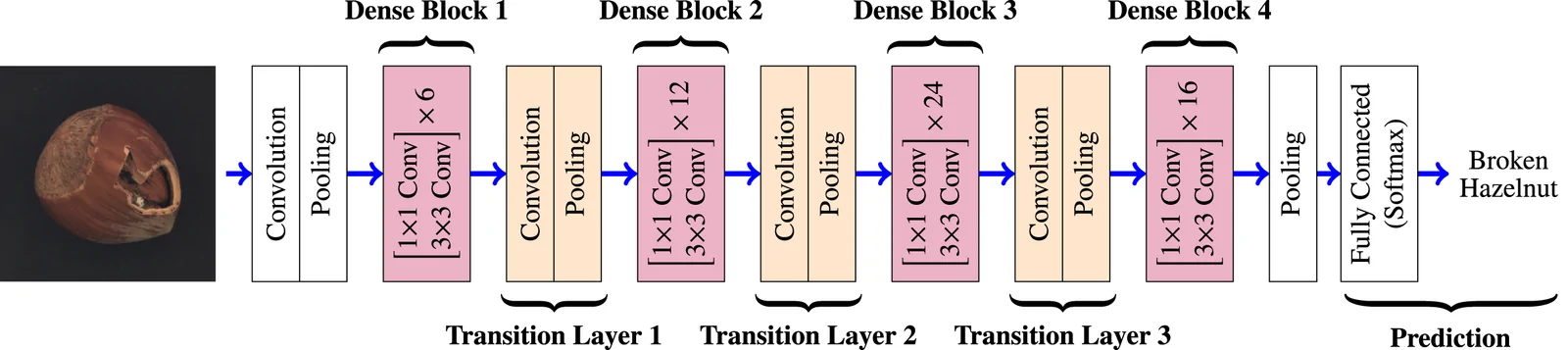
Architectural overview of DenseNet121, illustrating its densely connected convolutional blocks that enable feature reuse and enhance gradient flow efficiency.

## Materials and Methods

This section describes the proposed Dense121GAN framework, including the generator and discriminator architectures, the integration of DenseNet121, the adopted training strategy, and the evaluation protocol used for comparison with baseline models.

### Proposed method: Dense121GAN

Dense121GAN is a conditional Generative Adversarial Network (cGAN) designed for image-to-image translation and segmentation tasks. As illustrated in Fig. 2, the generator follows an encoder-decoder architecture in which a pretrained DenseNet121 encoder is integrated through transfer learning. All encoder layers remain frozen to preserve pretrained feature representations, improve training stability, and reduce computational cost, which is particularly beneficial for heterogeneous and limited-data datasets. Although freezing limits adaptation to dataset-specific features, the ablation study (‘Ablation Study’) showed that partial fine-tuning degraded performance on CVC-ClinicDB under the fixed training setting adopted in this work.

**Figure 2:**
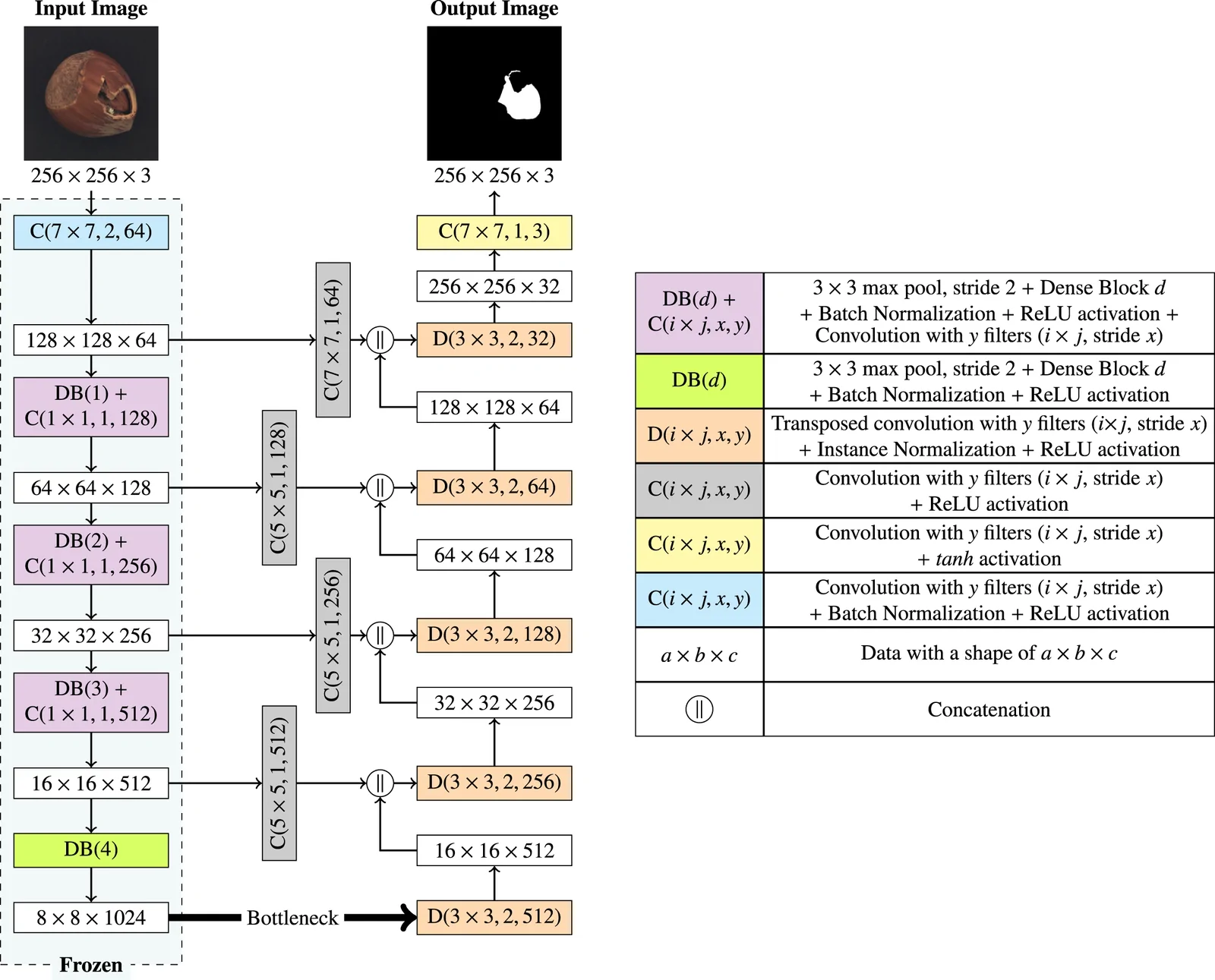
Architecture of the proposed Dense121GAN generator, integrating a pretrained DenseNet121 encoder with residual skip connections for improved feature propagation and reconstruction.

Four skip connections are extracted from different DenseNet121 dense blocks and connected to the decoder through convolutional layers and ReLU activation to preserve multi-scale spatial information during reconstruction. The decoder progressively upsamples bottleneck features using transposed convolutions until the original image resolution ( $256 \times 256 \times 3$) is restored. A final convolutional layer with *tanh* activation generates the output image.

The discriminator, illustrated in Fig. 3, follows the PatchGAN architecture, which evaluates image realism at the patch level. It consists of convolutional layers with increasing feature depth, instance normalization, and Leaky ReLU activation ( α=0.2), followed by a single-channel output layer for real/fake patch discrimination.

**Figure 3:**
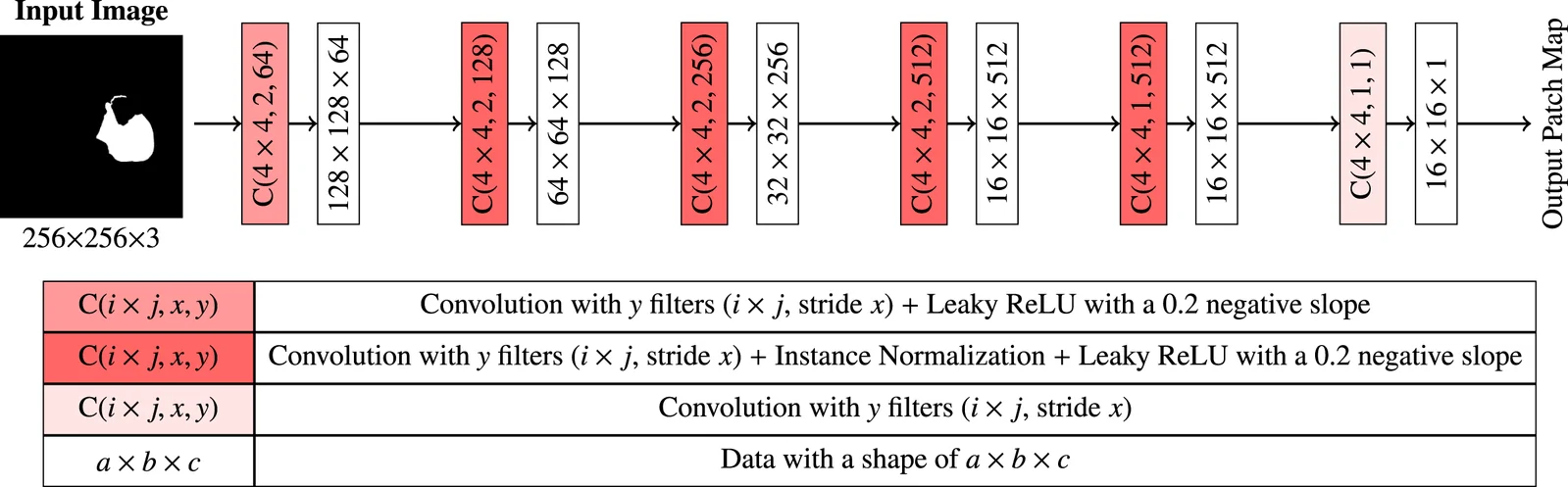
Architecture of the PatchGAN discriminator used to evaluate image realism at the patch level.

The generator is optimized using a weighted combination of adversarial and L1 losses:

(1) $${{\scr L}_G} = {{\scr L}_{\rm {GAN} }} + \lambda \cdot {{\scr L}_{\rm {L1} }},$$

where λ=100 balances perceptual realism and pixel-level reconstruction fidelity. The adversarial and L1 losses are defined as:

(2) $${{\scr L}_{\rm {GAN} }}(G,D) = {{\mathbb E}_{x,y}}[\log D(x,y)] + {{\mathbb E}_{x}}[\log (1 - D(x,G(x)))],$$

(3) $${{\scr L}_{\rm {L1} }}(G) = {{\mathbb E}_{x,y}}[||y - G(x)||{_1}].$$

Both generator and discriminator are optimized using Adam with ${\beta _{1}} = 0.5$. A linear learning-rate decay schedule is applied as follows:

(4) $${\rm {LR}}_{{\rm {epoch} }} = \left\{ {\matrix{ {{\eta _{0}},} \hfill & {{\mathrm{if\;}} e < {e_{\rm {decay} }},} \hfill \cr {{\eta _{0}} \cdot \left( {1 - {{e\ -\ {e_{\rm {decay} }}} \over {E\ -\ {e_{\rm {decay} }}}}} \right),} \hfill & {{\mathrm{otherwise}} ,} \hfill \cr } } \right.$$

where ${\eta _{0}}$ is the initial learning rate, e is the current epoch, ${e_{{{\mathrm{decay}}}}}$ is the decay starting epoch, and *E* is the total number of training epochs.

The model is quantitatively evaluated after each epoch, as described in ‘Evaluation Protocol’.

### Datasets

To evaluate the robustness and generalizability of the proposed model, experiments were conducted on multiple medical and industrial datasets with diverse segmentation characteristics.

1. **BCCD (Blood Cell Count and Detection)** (Depto et al., 2023, 2021; Anmol, 2018): A dataset of 1,328 microscopic blood smear images with corresponding segmentation masks. The original training/testing split was preserved for comparison with prior studies. For all remaining experiments, the subsets were merged and evaluated using the unified cross-validation protocol adopted in this work.
2. **Chest X-Ray (Montgomery and Shenzhen)** (Karmakar, 2022; Karmakar, 2020): A combined dataset of 704 posterior-anterior chest radiographs with lung segmentation masks, including both tuberculosis-positive and normal cases collected from the Montgomery and Shenzhen cohorts.
3. **CVC-ClinicDB** (Bernal et al., 2015; Al-Ghoniy, 2021): A polyp segmentation dataset containing 612 colonoscopy frames extracted from 29 video sequences, each paired with binary segmentation masks. Sequence-level splitting was applied during cross-validation to reduce temporal leakage between folds.
4. **BUSI (Breast Ultrasound Images Dataset)** (Al-Dhabyani et al., 2020; Shah, 2020): A breast ultrasound dataset containing 780 images with expert-annotated lesion masks. All benign, malignant, and normal categories were reformulated into a unified binary segmentation setting.
5. **Kvasir-SEG** (Jha et al., 2020; Kaggle, 2021a): A dataset of 1,000 endoscopic images with manually annotated polyp masks verified by expert gastroenterologists.
6. **Kvasir-Sessile** (Jha et al., 2020; Kaggle, 2021b): A challenging dataset containing 196 endoscopic images of sessile polyps with expert-annotated masks. The small size and subtle polyp structures make this dataset particularly difficult for segmentation models.
7. **MVTec AD** (Bergmann et al., 2019; Anmol, 2019): An industrial anomaly detection dataset. This study utilized ten categories: Bottle, Cable, Carpet, Hazelnut, Leather, Metal Nut, Pill, Toothbrush, Wood, and Zipper. Since the original dataset was designed for anomaly detection rather than supervised segmentation, the official training and testing subsets were merged and reformulated under the unified segmentation framework adopted in this study.

A standardized preprocessing pipeline was applied across all datasets. Images and masks were reorganized into a unified directory structure while preserving image-mask correspondence. All samples were resized to 256×256 pixels and stored in PNG format. Grayscale images were converted to RGB through channel replication to ensure compatibility with pretrained encoders expecting three-channel inputs. Channel replication preserves the original grayscale intensity distribution without introducing synthetic color information, thereby minimizing potential preprocessing bias while maintaining compatibility with pretrained RGB encoders.

To ensure fair comparison, all datasets were evaluated under a unified supervised segmentation framework using five-fold cross-validation. Splitting was performed before augmentation to prevent data leakage. In each fold, four subsets were used for training and one for testing. Final performance metrics were computed independently for each fold and reported as mean ± standard deviation.

Dataset-specific restructuring was applied when necessary. The original BCCD split was preserved only for comparison with prior studies, while sequence-level splitting was used for CVC-ClinicDB to prevent frames from the same colonoscopy sequence from appearing across different folds. For all remaining datasets, splitting was performed at the image level.

Special handling was required for MVTec AD because the official training split contains only defect-free images without segmentation masks. To enable supervised segmentation training and maintain consistent evaluation across all datasets, the official training and testing subsets were merged and evaluated using the same cross-validation protocol adopted throughout this work.

Data augmentation was applied only to industrial datasets, including Bottle, Cable, Carpet, Hazelnut, Leather, Metal Nut, Pill, Toothbrush, Wood, and Zipper. Augmentation included rotations and flips, producing an eightfold increase in training samples. No augmentation was applied to testing data. Medical datasets were intentionally excluded from augmentation to preserve pathological realism.

Finally, all compared models were trained and evaluated using identical data partitions and experimental settings to ensure fair comparison under a unified evaluation framework.

#### Ethics and data usage

All datasets used in this study are publicly available and anonymized. Therefore, no additional ethical approval or patient consent was required. All datasets were used in accordance with their respective licensing terms and citation requirements.

### Experiments and experimental setups

To evaluate the proposed Dense121GAN, comparative experiments were conducted using three generator architectures: UNet, ResNet-based, and Dense121GAN. The architectures of the UNet and ResNet generators are illustrated in Figs. 4 and 5. All models employed the same PatchGAN discriminator to ensure fair comparison.

**Figure 4:**
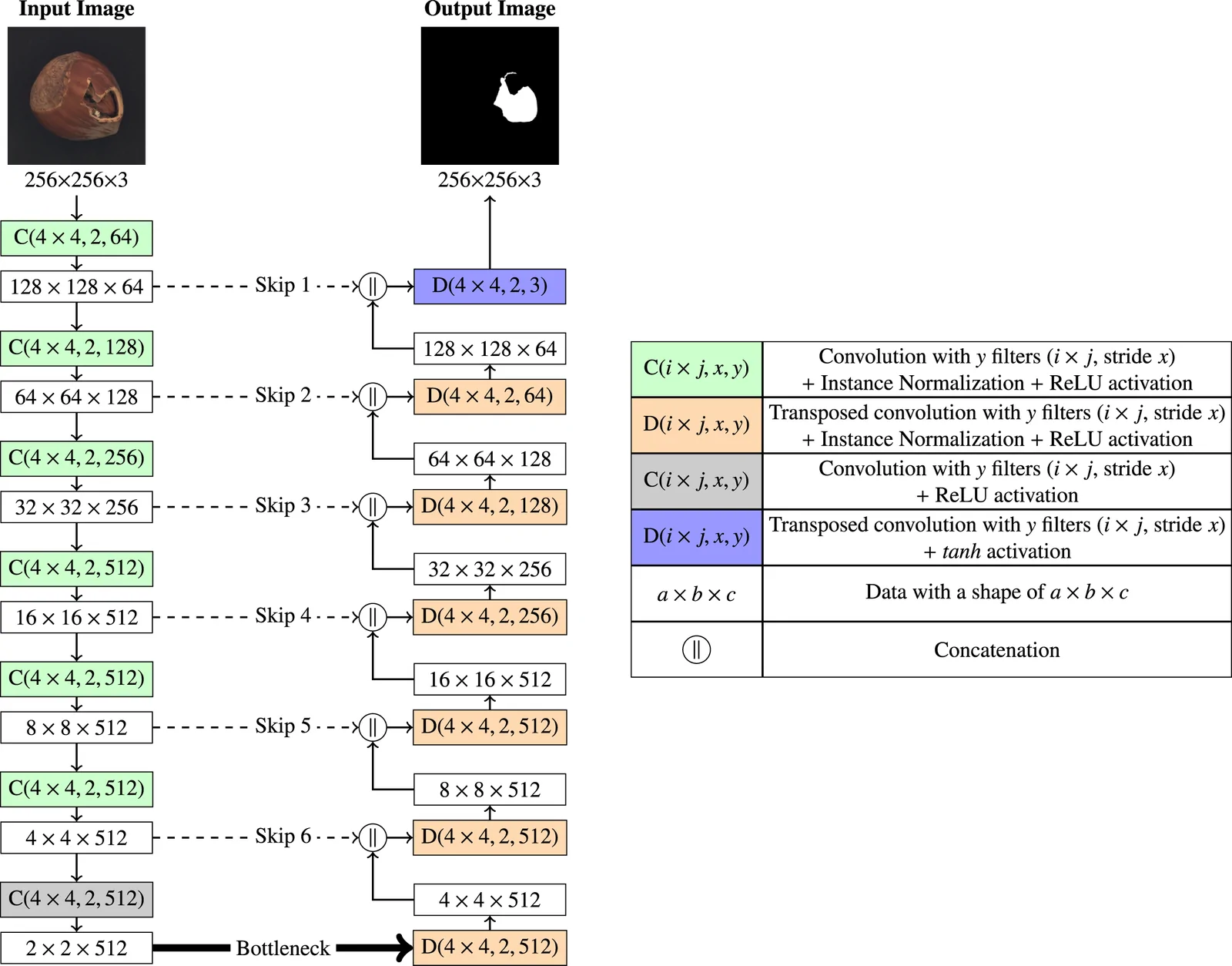
Architecture of the U-Net-based generator with encoder-decoder skip connections for spatial feature preservation.

**Figure 5:**
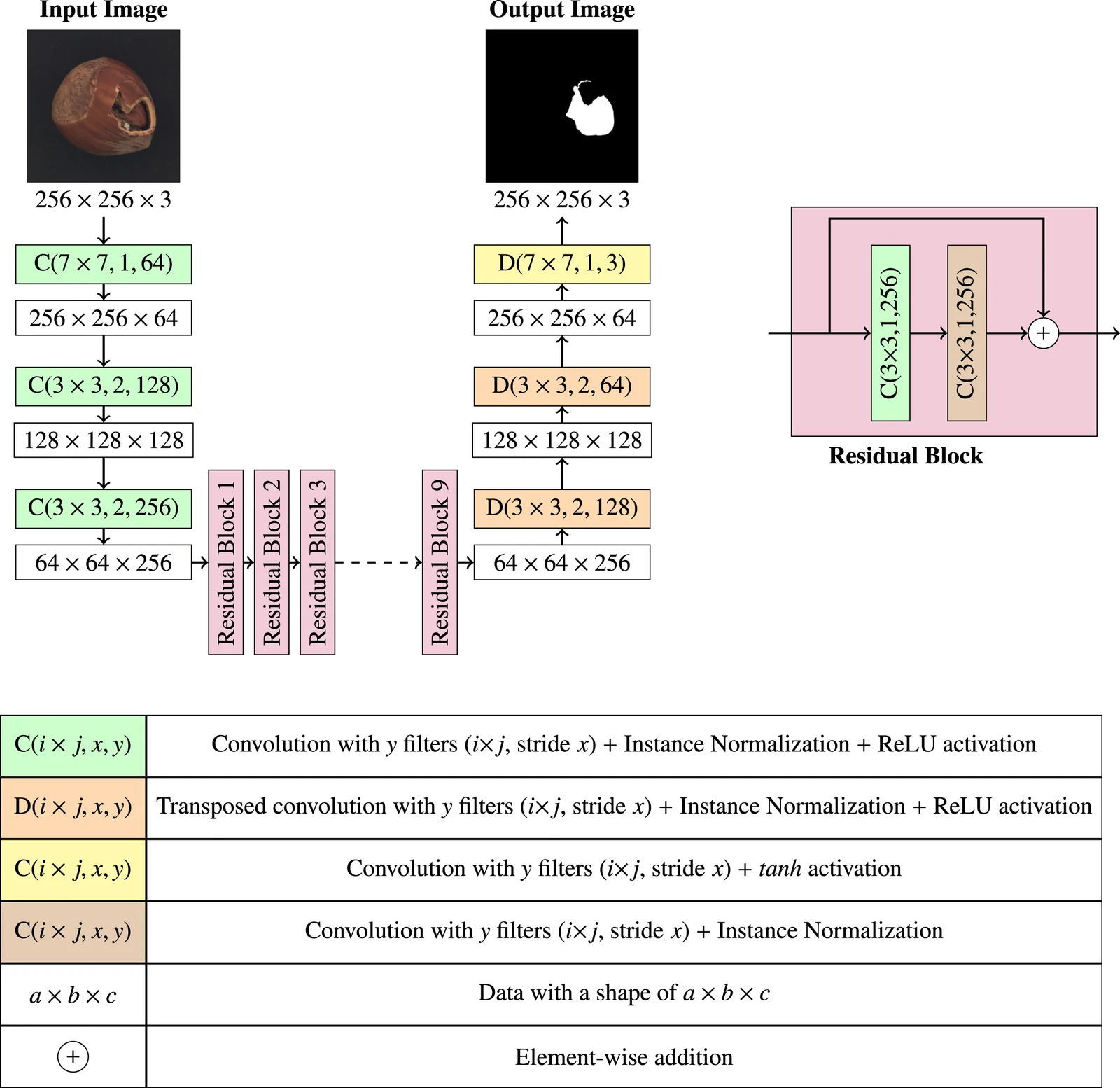
Architecture of the ResNet-based generator with residual blocks and transposed-convolution upsampling.

All generator-discriminator pairs were trained under identical conditions unless otherwise stated. Input and output images were resized to $256 \times 256 \times 3$ and normalized to the range [−1,1]. Training was performed using the Adam optimizer with an initial learning rate of ${\eta _{0}} = 2 \times {10^{ - 4}}$ and ${\beta _{1}} = 0.5$. All models were trained for 50 epochs with linear learning-rate decay applied from epoch 25 onward. The batch size was fixed at one following the original Pix2Pix configuration and GPU memory limitations.

The generator objective combined adversarial and L1 losses with λ=100 to balance perceptual realism and pixel-level reconstruction accuracy. Instance normalization was applied in both generator and discriminator networks to improve training stability. During optimization, the generator minimized the combined adversarial and reconstruction losses, while the discriminator learned to distinguish between real and generated image pairs under supervised paired training.

The implementation code, benchmark datasets, and experimental results are publicly available for evaluation and reproducibility in Davud (2025).

#### Implementation details and reproducibility

All experiments were implemented using TensorFlow 2.10.0 and Keras 2.10.0 under a unified training protocol across all models. Experiments were conducted on a Windows 10 system equipped with an NVIDIA GeForce RTX 4060 Laptop GPU (8 GB VRAM), an Intel CPU with 14 physical cores (20 logical cores), and 13.7 GB RAM, with CUDA acceleration enabled.

All models used identical optimizer settings, learning rates, loss functions, and training durations to ensure fair comparison. Rather than fixing a random seed, robustness and reliability were evaluated through stratified five-fold cross-validation, with final results reported as the average performance across folds. All implementation resources and experimental files are publicly available in Davud (2025).

#### Evaluation protocol

Testing was performed at the end of each epoch within every fold to record evaluation metrics and monitor training dynamics. These intermediate results were also used for convergence analysis and visualization of model behavior across training epochs, as illustrated in ‘Results’. The testing process had no influence on model optimization, and no validation set was used for early stopping or model selection.

All models were trained for a fixed 50 epochs under identical training conditions. Final reported results correspond to epoch 50 and were averaged across the five cross-validation folds to ensure fair comparison between Dense121GAN, UNet, and ResNet-based generators.

#### Evaluation metrics

To comprehensively evaluate segmentation and reconstruction performance, multiple complementary metrics were employed. Segmentation quality was assessed using Intersection over Union (IoU), Dice coefficient (Dice), and specificity (SPEC). To provide more balanced evaluation under class imbalance, Matthews Correlation Coefficient (MCC), negative predictive value (NPV), and Cohen’s Kappa were additionally included.

Reconstruction fidelity was evaluated using mean squared error (MSE), structural similarity index measure (SSIM), coefficient of determination ( ${R^{2}}$), and symmetric mean absolute percentage error (SMAPE).

Higher values of IoU, Dice, SPEC, MCC, NPV, Kappa, SSIM, and ${R^{2}}$ indicate better performance, whereas lower values of MSE and SMAPE indicate reduced reconstruction error. Using multiple complementary metrics provides a more robust assessment of segmentation accuracy, reconstruction fidelity, and robustness under class imbalance.

## Results

### Quantitative results

Tables 1 and 2 present the evaluation results at epoch 50 across multiple publicly available datasets, including BCCD (Depto et al., 2023, 2021), Chest X-Ray (Karmakar, 2022), CVC-ClinicDB (Bernal et al., 2015), BUSI (Al-Dhabyani et al., 2020), Kvasir-SEG and Kvasir-Sessile (Jha et al., 2020), and MVTec AD categories (Bergmann et al., 2019). Table 1 summarizes segmentation and classification metrics, including IoU, Dice, SPEC, MCC, NPV, and Kappa. Table 2 reports regression and perceptual quality metrics, including MSE, SSIM, ${R^{2}}$, and SMAPE. The inclusion of MCC, SPEC, NPV, and Kappa supports binary classification assessment at the pixel level, while ${R^{2}}$ and SMAPE provide complementary regression-based evaluation of pixel-intensity reconstruction. Bold values indicate the best performance for each metric and dataset among the compared models.

**Table 1:** Segmentation and classification performance of generator models at epoch 50 across the evaluated datasets. Results are reported as mean ± standard deviation over five-fold cross-validation. Bold values indicate the best performance for each metric and dataset among the compared models.

Dataset	Technique	IoU	Dice	SPEC	MCC	NPV	Kappa
**BCCD**	Dense121GAN	0.9289 ± 0.0029	0.9628 ± 0.0016	0.9813 ± 0.0012	0.9458 ± 0.0021	0.9839 ± 0.0005	0.9452 ± 0.0022
	ResNet	**0.9327 ± 0.0030**	**0.9648 ± 0.0017**	**0.9820 ± 0.0012**	**0.9487 ± 0.0021**	**0.9849 ± 0.0004**	**0.9482 ± 0.0022**
	UNet	0.9274 ± 0.0030	0.9619 ± 0.0017	0.9794 ± 0.0012	0.9445 ± 0.0023	0.9846 ± 0.0005	0.9438 ± 0.0024
**Chest X-Ray**	Dense121GAN	0.9225 ± 0.0033	0.9589 ± 0.0020	0.9878 ± 0.0011	0.9461 ± 0.0023	0.9850 ± 0.0019	0.9453 ± 0.0025
	ResNet	**0.9283 ± 0.0033**	**0.9621 ± 0.0019**	**0.9896 ± 0.0007**	**0.9503 ± 0.0023**	**0.9854 ± 0.0017**	**0.9495 ± 0.0024**
	UNet	0.9213 ± 0.0013	0.9581 ± 0.0008	0.9886 ± 0.0013	0.9453 ± 0.0009	0.9840 ± 0.0016	0.9443 ± 0.0009
**CVC-ClinicDB**	Dense121GAN	**0.8411 ± 0.0218**	**0.9034 ± 0.0161**	0.9944 ± 0.0008	**0.8996 ± 0.0157**	**0.9894 ± 0.0053**	**0.8956 ± 0.0172**
	ResNet	0.0000 ± 0.0000	0.0000 ± 0.0000	**1.0000 ± 0.0000**	0.0000 ± 0.0000	0.9084 ± 0.0081	0.0000 ± 0.0000
	UNet	0.7551 ± 0.0256	0.8337 ± 0.0277	0.9922 ± 0.0019	0.8302 ± 0.0266	0.9829 ± 0.0044	0.8223 ± 0.0274
**BUSI**	Dense121GAN	**0.5457 ± 0.0213**	**0.6220 ± 0.0237**	0.9831 ± 0.0034	**0.6161 ± 0.0200**	**0.9744 ± 0.0047**	**0.6048 ± 0.0215**
	ResNet	0.0000 ± 0.0000	0.0000 ± 0.0000	**1.0000 ± 0.0000**	0.0000 ± 0.0000	0.9217 ± 0.0117	0.0000 ± 0.0000
	UNet	0.4834 ± 0.0214	0.5600 ± 0.0212	0.9824 ± 0.0035	0.5585 ± 0.0182	0.9711 ± 0.0069	0.5433 ± 0.0205
**Kvasir-SEG**	Dense121GAN	**0.7582 ± 0.0149**	**0.8411 ± 0.0129**	0.9779 ± 0.0027	**0.8275 ± 0.0117**	**0.9655 ± 0.0061**	**0.8145 ± 0.0114**
	ResNet	0.0000 ± 0.0000	0.0000 ± 0.0000	**1.0000 ± 0.0000**	0.0000 ± 0.0000	0.8461 ± 0.0119	0.0000 ± 0.0000
	UNet	0.5408 ± 0.0215	0.6479 ± 0.0230	0.9622 ± 0.0052	0.6325 ± 0.0240	0.9329 ± 0.0109	0.6051 ± 0.0253
**Kvasir-Sessile**	Dense121GAN	**0.5621 ± 0.0565**	**0.6907 ± 0.0507**	0.9794 ± 0.0051	**0.6880 ± 0.0528**	**0.9662 ± 0.0094**	**0.6665 ± 0.0559**
	ResNet	0.0000 ± 0.0000	0.0000 ± 0.0000	**1.0000 ± 0.0000**	0.0000 ± 0.0000	0.9231 ± 0.0124	0.0000 ± 0.0000
	UNet	0.0148 ± 0.0323	0.0226 ± 0.0492	0.9958 ± 0.0093	0.0208 ± 0.0429	0.9245 ± 0.0142	0.0180 ± 0.0389
**Bottle**	Dense121GAN	**0.5900 ± 0.1331**	**0.6566 ± 0.1436**	0.9941 ± 0.0037	**0.6551 ± 0.1436**	**0.9923 ± 0.0029**	**0.6500 ± 0.1430**
	ResNet	0.0000 ± 0.0000	0.0000 ± 0.0000	**1.0000 ± 0.0000**	0.0000 ± 0.0000	0.9421 ± 0.0131	0.0000 ± 0.0000
	UNet	0.4524 ± 0.1045	0.5382 ± 0.1191	0.9928 ± 0.0023	0.5376 ± 0.1166	0.9813 ± 0.0065	0.5269 ± 0.1183
**Cable**	Dense121GAN	**0.3575 ± 0.0258**	**0.4180 ± 0.0248**	0.9968 ± 0.0009	**0.4247 ± 0.0219**	**0.9906 ± 0.0038**	**0.4138 ± 0.0250**
	ResNet	0.0000 ± 0.0000	0.0000 ± 0.0000	**1.0000 ± 0.0000**	0.0000 ± 0.0000	0.9713 ± 0.0036	0.0000 ± 0.0000
	UNet	0.1799 ± 0.1635	0.2255 ± 0.2034	0.9957 ± 0.0040	0.2304 ± 0.2030	0.9807 ± 0.0118	0.2211 ± 0.1996
**Carpet**	Dense121GAN	**0.5043 ± 0.0688**	**0.5970 ± 0.0845**	0.9981 ± 0.0005	**0.6001 ± 0.0831**	**0.9968 ± 0.0015**	**0.5945 ± 0.0837**
	ResNet	0.1746 ± 0.2420	0.2098 ± 0.2905	**0.9992 ± 0.0011**	0.2121 ± 0.2937	0.9884 ± 0.0086	0.2088 ± 0.2892
	UNet	0.4672 ± 0.0681	0.5644 ± 0.0801	0.9981 ± 0.0007	0.5714 ± 0.0804	0.9962 ± 0.0014	0.5617 ± 0.0795
**Hazelnut**	Dense121GAN	**0.5071 ± 0.0530**	**0.5612 ± 0.0547**	0.9989 ± 0.0005	**0.5616 ± 0.0541**	0.9969 ± 0.0027	**0.5591 ± 0.0545**
	ResNet	0.0000 ± 0.0000	0.0000 ± 0.0000	**1.0000 ± 0.0000**	0.0000 ± 0.0000	0.9787 ± 0.0126	0.0000 ± 0.0000
	UNet	0.4925 ± 0.0563	0.5491 ± 0.0547	0.9983 ± 0.0011	0.5514 ± 0.0540	**0.9972 ± 0.0012**	0.5470 ± 0.0544
**Leather**	Dense121GAN	**0.4798 ± 0.0581**	**0.5745 ± 0.0646**	0.9991 ± 0.0007	**0.5822 ± 0.0643**	**0.9983 ± 0.0008**	**0.5733 ± 0.0644**
	ResNet	0.0845 ± 0.1889	0.1011 ± 0.2260	**0.9999 ± 0.0002**	0.1018 ± 0.2277	0.9940 ± 0.0036	0.1009 ± 0.2257
	UNet	0.4597 ± 0.0455	0.5547 ± 0.0540	0.9987 ± 0.0004	0.5656 ± 0.0559	0.9980 ± 0.0014	0.5532 ± 0.0538
**Metal nut**	Dense121GAN	**0.6267 ± 0.0987**	**0.6949 ± 0.0951**	0.9961 ± 0.0016	**0.6951 ± 0.0951**	**0.9937 ± 0.0017**	**0.6900 ± 0.0946**
	ResNet	0.0000 ± 0.0000	0.0000 ± 0.0000	**1.0000 ± 0.0000**	0.0000 ± 0.0000	0.8828 ± 0.0557	0.0000 ± 0.0000
	UNet	0.5957 ± 0.0941	0.6668 ± 0.0925	0.9964 ± 0.0016	0.6691 ± 0.0910	0.9925 ± 0.0012	0.6616 ± 0.0925
**Pill**	Dense121GAN	**0.5597 ± 0.0646**	**0.6514 ± 0.0655**	0.9984 ± 0.0005	**0.6579 ± 0.0632**	**0.9967 ± 0.0011**	**0.6491 ± 0.0654**
	ResNet	0.0000 ± 0.0000	0.0000 ± 0.0000	**1.0000 ± 0.0000**	0.0000 ± 0.0000	0.9663 ± 0.0205	0.0000 ± 0.0000
	UNet	0.0983 ± 0.0450	0.1212 ± 0.0481	0.9995 ± 0.0005	0.1331 ± 0.0467	0.9829 ± 0.0097	0.1184 ± 0.0481
**Toothbrush**	Dense121GAN	**0.2969 ± 0.1156**	**0.3880 ± 0.1390**	0.9972 ± 0.0014	**0.3987 ± 0.1339**	**0.9941 ± 0.0039**	**0.3842 ± 0.1385**
	ResNet	0.0000 ± 0.0000	0.0000 ± 0.0000	**1.0000 ± 0.0000**	0.0000 ± 0.0000	0.9846 ± 0.0074	0.0000 ± 0.0000
	UNet	0.1456 ± 0.0616	0.1956 ± 0.0929	0.9976 ± 0.0019	0.2073 ± 0.1055	0.9909 ± 0.0058	0.1919 ± 0.0916
**Wood**	Dense121GAN	**0.5226 ± 0.0525**	**0.6130 ± 0.0580**	0.9957 ± 0.0011	**0.6116 ± 0.0572**	**0.9890 ± 0.0072**	**0.6056 ± 0.0568**
	ResNet	0.0000 ± 0.0000	0.0000 ± 0.0000	**1.0000 ± 0.0000**	0.0000 ± 0.0000	0.9611 ± 0.0163	0.0000 ± 0.0000
	UNet	0.5039 ± 0.0519	0.5979 ± 0.0601	0.9965 ± 0.0005	0.5988 ± 0.0574	0.9873 ± 0.0068	0.5903 ± 0.0580
**Zipper**	Dense121GAN	**0.5254 ± 0.0407**	**0.6240 ± 0.0465**	0.9965 ± 0.0002	**0.6270 ± 0.0487**	**0.9958 ± 0.0007**	**0.6203 ± 0.0469**
	ResNet	0.0000 ± 0.0000	0.0000 ± 0.0000	**1.0000 ± 0.0000**	0.0000 ± 0.0000	0.9793 ± 0.0037	0.0000 ± 0.0000
	UNet	0.4583 ± 0.0429	0.5694 ± 0.0455	0.9969 ± 0.0007	0.5754 ± 0.0463	0.9933 ± 0.0013	0.5647 ± 0.0460

**Table 2:** Regression and perceptual quality evaluation of generator models at epoch 50 across the evaluated datasets. Results are reported as mean ± standard deviation over five-fold cross-validation. Bold values denote the best performance for each metric and dataset among the compared models.

Dataset	Technique	MSE	SSIM	${R^{2}}$	SMAPE
**BCCD**	Dense121GAN	0.0115 ± 0.0006	0.9270 ± 0.0030	0.9391 ± 0.0033	0.2280 ± 0.0046
	ResNet	**0.0106 ± 0.0005**	**0.9328 ± 0.0028**	**0.9436 ± 0.0035**	0.2296 ± 0.0016
	UNet	0.0119 ± 0.0006	0.9243 ± 0.0029	0.9364 ± 0.0038	**0.2141 ± 0.0029**
**Chest X-Ray**	Dense121GAN	0.0162 ± 0.0009	0.9299 ± 0.0009	0.9115 ± 0.0050	0.0918 ± 0.0017
	ResNet	**0.0147 ± 0.0009**	**0.9366 ± 0.0013**	**0.9198 ± 0.0048**	**0.0815 ± 0.0010**
	UNet	0.0163 ± 0.0004	0.9297 ± 0.0006	0.9097 ± 0.0024	0.0961 ± 0.0019
**CVC-ClinicDB**	Dense121GAN	**0.0124 ± 0.0044**	**0.9604 ± 0.0065**	**0.8003 ± 0.0446**	**0.0542 ± 0.0094**
	ResNet	0.0907 ± 0.0081	0.8827 ± 0.0099	−0.1115 ± 0.0123	0.2003 ± 0.0178
	UNet	0.0201 ± 0.0039	0.9468 ± 0.0049	0.6568 ± 0.0980	0.0755 ± 0.0081
**BUSI**	Dense121GAN	**0.0352 ± 0.0011**	**0.9307 ± 0.0029**	**0.2844 ± 0.1389**	**0.0998 ± 0.0031**
	ResNet	0.0775 ± 0.0116	0.9019 ± 0.0136	−0.1016 ± 0.0193	0.1682 ± 0.0244
	UNet	0.0393 ± 0.0058	0.9262 ± 0.0075	0.1400 ± 0.1658	0.1116 ± 0.0121
**Kvasir-SEG**	Dense121GAN	**0.0418 ± 0.0018**	**0.9091 ± 0.0024**	**0.4917 ± 0.0972**	**0.1384 ± 0.0041**
	ResNet	0.1527 ± 0.0119	0.8152 ± 0.0126	−0.2298 ± 0.0315	0.3281 ± 0.0240
	UNet	0.0866 ± 0.0082	0.8526 ± 0.0086	−0.0633 ± 0.1861	0.2499 ± 0.0130
**Kvasir-sessile**	Dense121GAN	**0.0458 ± 0.0117**	**0.9046 ± 0.0182**	**0.1996 ± 0.1532**	**0.1645 ± 0.0294**
	ResNet	0.0761 ± 0.0124	0.9022 ± 0.0143	−0.0984 ± 0.0206	0.1745 ± 0.0258
	UNet	0.0770 ± 0.0114	0.8888 ± 0.0124	−0.1676 ± 0.1590	0.3835 ± 0.0837
**Bottle**	Dense121GAN	**0.0108 ± 0.0024**	**0.9652 ± 0.0084**	**0.5689 ± 0.1226**	**0.0426 ± 0.0097**
	ResNet	0.0571 ± 0.0129	0.9207 ± 0.0188	−0.0672 ± 0.0163	0.1294 ± 0.0297
	UNet	0.0218 ± 0.0061	0.9476 ± 0.0139	0.3904 ± 0.0824	0.0738 ± 0.0174
**Cable**	Dense121GAN	**0.0111 ± 0.0036**	**0.9713 ± 0.0064**	**0.3201 ± 0.0329**	**0.0444 ± 0.0089**
	ResNet	0.0281 ± 0.0035	0.9558 ± 0.0045	−0.0319 ± 0.0048	0.0679 ± 0.0075
	UNet	0.0219 ± 0.0089	0.9561 ± 0.0055	0.1100 ± 0.1318	0.0675 ± 0.0119
**Carpet**	Dense121GAN	**0.0043 ± 0.0016**	**0.9846 ± 0.0042**	**0.4953 ± 0.0617**	**0.0188 ± 0.0045**
	ResNet	0.0118 ± 0.0077	0.9775 ± 0.0084	0.1526 ± 0.2406	0.0319 ± 0.0151
	UNet	0.0048 ± 0.0014	0.9831 ± 0.0040	0.4534 ± 0.0731	0.0237 ± 0.0042
**Hazelnut**	Dense121GAN	**0.0030 ± 0.0018**	**0.9866 ± 0.0042**	**0.5239 ± 0.0507**	**0.0198 ± 0.0043**
	ResNet	0.0208 ± 0.0125	0.9656 ± 0.0144	−0.0251 ± 0.0166	0.0519 ± 0.0259
	UNet	0.0034 ± 0.0008	0.9846 ± 0.0030	0.4847 ± 0.0589	0.0227 ± 0.0028
**Leather**	Dense121GAN	**0.0021 ± 0.0010**	**0.9918 ± 0.0029**	**0.4462 ± 0.0765**	**0.0098 ± 0.0034**
	ResNet	0.0058 ± 0.0034	0.9882 ± 0.0052	0.0814 ± 0.1992	0.0163 ± 0.0078
	UNet	0.0027 ± 0.0013	0.9903 ± 0.0033	0.3515 ± 0.0803	0.0130 ± 0.0036
**Metal Nut**	Dense121GAN	**0.0069 ± 0.0017**	**0.9717 ± 0.0041**	**0.6090 ± 0.0903**	**0.0405 ± 0.0053**
	ResNet	0.1161 ± 0.0554	0.8530 ± 0.0648	−0.2110 ± 0.1130	0.2521 ± 0.1161
	UNet	0.0077 ± 0.0015	0.9700 ± 0.0041	0.5849 ± 0.0945	0.0458 ± 0.0062
**Pill**	Dense121GAN	**0.0039 ± 0.0011**	**0.9856 ± 0.0028**	**0.5203 ± 0.1506**	**0.0190 ± 0.0037**
	ResNet	0.0333 ± 0.0204	0.9539 ± 0.0236	−0.0501 ± 0.0346	0.0751 ± 0.0425
	UNet	0.0155 ± 0.0075	0.9656 ± 0.0161	0.0322 ± 0.0817	0.1512 ± 0.0761
**Toothbrush**	Dense121GAN	**0.0071 ± 0.0034**	**0.9750 ± 0.0064**	**0.2716 ± 0.1178**	**0.0356 ± 0.0069**
	ResNet	0.0146 ± 0.0074	0.9701 ± 0.0095	−0.0172 ± 0.0085	0.0416 ± 0.0159
	UNet	0.0101 ± 0.0055	0.9721 ± 0.0090	0.0766 ± 0.0931	0.0675 ± 0.0129
**Wood**	Dense121GAN	**0.0120 ± 0.0062**	**0.9613 ± 0.0120**	**0.4973 ± 0.0589**	**0.0506 ± 0.0141**
	ResNet	0.0379 ± 0.0161	0.9383 ± 0.0204	−0.0479 ± 0.0244	0.0939 ± 0.0354
	UNet	0.0129 ± 0.0050	0.9594 ± 0.0101	0.4884 ± 0.0491	0.0563 ± 0.0115
**Zipper**	Dense121GAN	**0.0066 ± 0.0007**	**0.9775 ± 0.0015**	**0.4675 ± 0.0507**	**0.0310 ± 0.0013**
	ResNet	0.0202 ± 0.0037	0.9678 ± 0.0044	−0.0220 ± 0.0041	0.0493 ± 0.0078
	UNet	0.0087 ± 0.0013	0.9750 ± 0.0025	0.3907 ± 0.0685	0.0326 ± 0.0036

Dense121GAN outperformed both UNet and ResNet in the majority of datasets (14 out of 16) under the unified experimental setting, demonstrating consistent performance across diverse segmentation tasks. In the remaining two datasets, namely BCCD and Chest X-Ray, Dense121GAN achieved performance comparable to ResNet while still outperforming UNet.

To further analyze the training dynamics, the convergence behavior of Dense121GAN and UNet was evaluated using IoU values recorded throughout training, as illustrated in Fig. 6. The convergence epoch was defined as the first epoch at which the IoU reached the final performance range, determined as the mean ± standard deviation of the IoU at epoch 50 across all folds. In cases where models exhibited unstable behavior, such as mode collapse or highly fluctuating IoU trajectories, convergence could not be reliably determined. Therefore, convergence analysis was reported only for datasets in which a meaningful comparison between Dense121GAN and at least one non-collapsed baseline model could be established.

**Figure 6:**
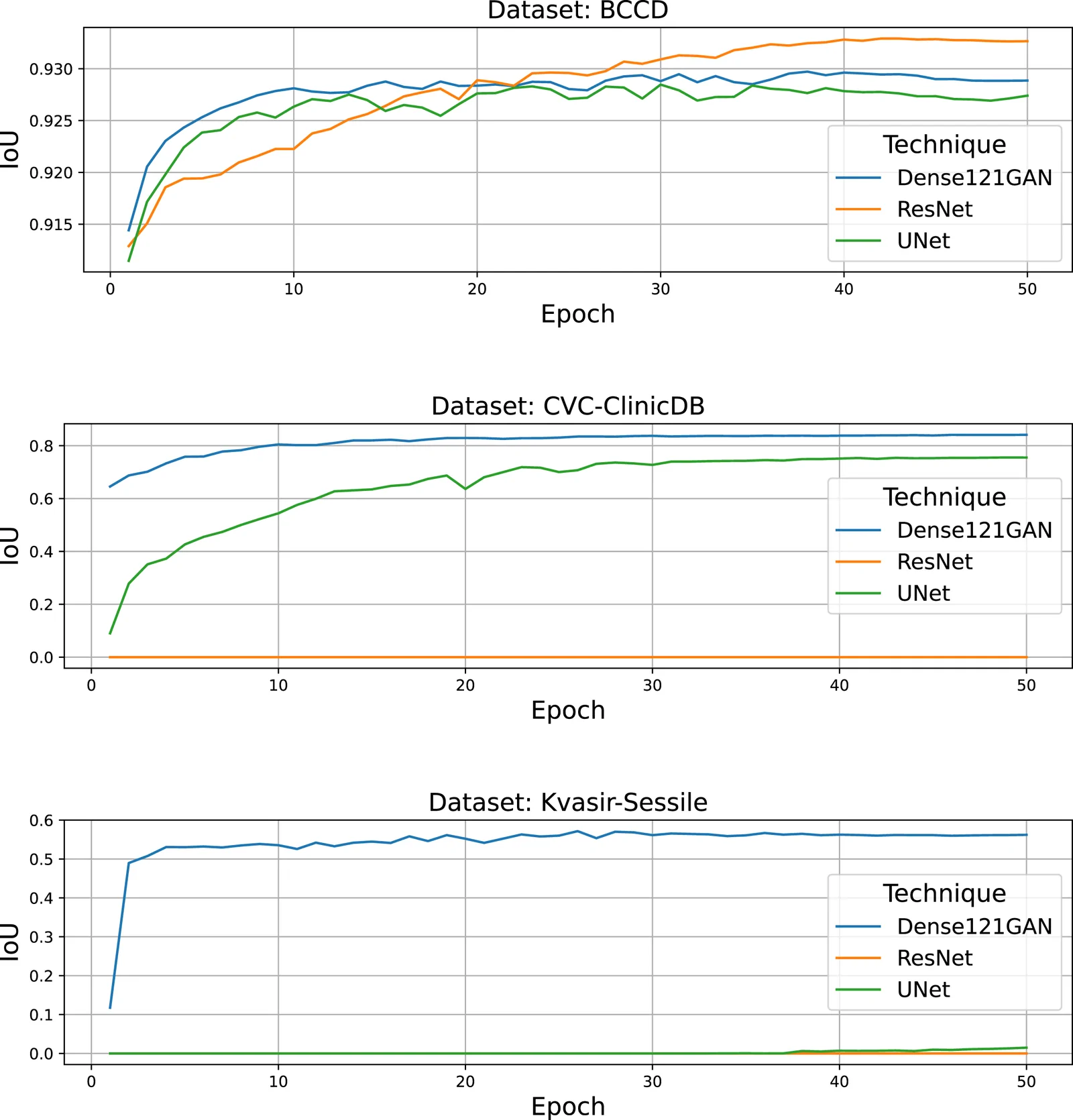
Representative convergence patterns of average Intersection over Union (IoU) over 50 training epochs for different datasets and segmentation models. Similar optimization behaviors were observed across other datasets following the same convergence trends.

Figure 6 presents representative IoU convergence patterns observed across the evaluated datasets. BCCD and Chest-X-Ray showed similar behavior, with ResNet achieving slightly higher final IoU values. A second convergence pattern, observed in datasets including CVC-ClinicDB, BUSI, Bottle, Cable, Carpet, Hazelnut, Kvasir-SEG, Leather, Metal Nut, Toothbrush, Wood, and Zipper, demonstrated superior and more stable convergence for Dense121GAN, followed by UNet, whereas ResNet suffered from unstable optimization. Finally, Kvasir-Sessile and Pill represented the most challenging optimization scenario, where both UNet and ResNet collapsed while Dense121GAN maintained stable convergence. The plotted curves represent the average IoU values across the five testing folds evaluated after each epoch without affecting the training process, thereby ensuring unbiased performance monitoring throughout training.

Table 3 summarizes the convergence epochs determined from recorded IoU values across datasets. Dense121GAN consistently reached stable performance earlier than UNet, requiring 5–68% fewer training epochs, with an average reduction of 44%. These convergence measurements indicate earlier stabilization behavior of Dense121GAN across datasets of varying difficulty. In addition to faster convergence, Dense121GAN generally achieved superior performance across most evaluation metrics while maintaining competitive results in more challenging cases.

**Table 3:** Convergence epochs determined from recorded IoU values.

Dataset	Dense121GAN	UNet	Reduction (%)
BCCD	5	6	17
Chest X-ray	4	6	33
CVC-ClinicDB	10	18	44
BUSI	12	25	52
Kvasir-SEG	6	10	40
Bottle	12	28	57
Cable	8	25	68
Carpet	15	30	50
Hazelnut	12	28	57
Leather	18	30	40
Metal nut	15	28	46
Toothbrush	40	42	5
Wood	17	31	45
Zipper	7	21	67
**Minimum**	–	–	**5**
**Maximum**	–	–	**68**
**Average**	–	–	**44**

While convergence behavior reflects training dynamics, computational efficiency also plays an important role in evaluating segmentation models. Table 4 presents the computational characteristics of the trained generator models under identical input conditions ( $256 \times 256 \times 3$), including parameter counts, model size, and peak GPU memory usage during inference.

**Table 4:** Computational efficiency comparison of trained generator models.

Model	Total params (M)	Trainable params (M)	Frozen params (M)	Model size (MB)	Peak GPU memory (GB)
Dense121GAN	23.70	16.66	7.04	218.78	0.18
UNet	41.83	41.83	0.00	478.98	0.34
ResNet	11.39	11.39	0.00	130.74	0.39

Dense121GAN contains fewer total parameters than UNet (23.70M *vs*. 41.83M) and significantly fewer trainable parameters due to its frozen DenseNet121 encoder. It also requires substantially lower peak GPU memory (0.18 GB) compared to both UNet (0.34 GB) and ResNet (0.39 GB). Although the ResNet-based generator has the lowest parameter count, it exhibited instability and mode collapse across multiple datasets, resulting in poor segmentation performance. These observations indicate that parameter efficiency alone is insufficient for reliable segmentation.

The convergence trends observed in Fig. 6 further confirm that Dense121GAN maintains stable and consistent learning behavior throughout training. In contrast, ResNet frequently exhibited mode collapse, while UNet showed instability in challenging datasets, particularly those involving small or subtle defects.

### Comparison with state-of-the-art methods

Table 5 compares the performance of Dense121GAN with state-of-the-art segmentation models across multiple datasets. The reported results were extracted from the corresponding studies. For the BCCD dataset, Dense121GAN was evaluated using the original training and testing split to ensure fair comparison with prior works. For the remaining datasets, results were obtained using the proposed five-fold cross-validation protocol, as standardized predefined splits were not consistently available across studies. Bold values indicate the best reported performance for each metric and dataset, while the symbol “–” denotes metrics not reported in the corresponding studies.

**Table 5:** Comparison of Dense121GAN with state-of-the-art segmentation models across multiple datasets. Bold values highlight the best performance for each metric and dataset among the compared models. The symbol “–” indicates that the corresponding metric was not reported in the respective study. In this table, results for the BCCD dataset were obtained using the original training and testing split to ensure fair comparison with prior studies. For the remaining datasets, results were obtained using the proposed five-fold cross-validation protocol, as predefined training and testing splits were not consistently available.

Dataset	Model	Accuracy	Dice (%)	IoU (%)
BCCD (Depto et al., 2023, 2021)	Retinex U-Net (Chen & Zhu, 2022)	0.8833	0.8040	0.7648
	CellSegUNet (Metlek, 2024)	0.9250	0.8990	0.8850
	DeepLabV3+ (Toptaş & Hanbay, 2023)	0.9428	0.9429	0.8957
	Dense121GAN (proposed)	**0.9691**	**0.9527**	**0.9160**
Chest X-Ray (Karmakar, 2022)	Souza et al. (2019)	0.9697	0.9356	0.8807
	TB-UNet (Iqbal, Usman & Ahmed, 2023)	0.9770	–	0.8168
	DEFUNet (Sharma et al., 2022)	0.9635	0.9488	0.9038
	Bhattacharya et al. (2023), Kamil & Hashem (2022)	0.9147	–	0.7494
	FCN (U-Net) (Rashid, Akram & Hassan, 2018)	0.9710	0.9510	–
	Dense121GAN (proposed)	**0.9798**	**0.9589**	**0.9225**
CVC-ClinicDB (Bernal et al., 2015)	Bhattacharya et al. (2023)	–	0.5400	0.4500
	FCBFormer+SwinV2 (Fitzgerald & Matuszewski, 2023)	–	0.9001	0.8261
	Xcep-MResUNet (Aghalari & Bizaki, 2024)	–	0.8042	0.7327
	Fang et al. (2019)	0.9668	0.8308	0.7623
	Dense121GAN (proposed)	**0.9859**	**0.9034**	**0.8411**
BUSI (Al-Dhabyani et al., 2020)	Anari et al. (2025)	–	0.6140	0.5305
	Dense121GAN (proposed)	**0.9628**	**0.6220**	**0.5457**
Kvasir-SEG (Jha et al., 2020)	Bhattacharya et al. (2023)	–	0.7500	0.6500
	FCBFormer+SwinV2 (Fitzgerald & Matuszewski, 2023)	–	**0.9420**	**0.8973**
	Xcep-MResUNet (Aghalari & Bizaki, 2024)	–	0.8890	0.8249
	Dense121GAN (proposed)	**0.9546**	0.8411	0.7582

Despite differences in experimental protocols, Dense121GAN demonstrated superior or competitive performance across several medical imaging datasets, including BCCD, BUSI, Chest X-Ray, and CVC-ClinicDB, particularly in terms of Dice coefficient and IoU. Notably, the MVTec AD categories were originally designed for anomaly detection rather than segmentation, and to the best of our knowledge, no directly comparable segmentation-based studies are currently available for these datasets.

Finally, comparison with state-of-the-art models further demonstrates the effectiveness of Dense121GAN across diverse segmentation tasks. For the BCCD dataset, comparisons were performed using the original training and testing split to ensure consistency with prior studies. For the remaining datasets, Dense121GAN was evaluated using the proposed unified cross-validation protocol, while previously reported works may follow different experimental settings and dataset partitions. Therefore, these comparisons should be interpreted as contextual rather than strictly one-to-one evaluations. Nevertheless, Dense121GAN achieved superior or competitive performance across multiple medical imaging benchmarks, including BCCD, BUSI, Chest X-Ray, and CVC-ClinicDB, supporting its robustness and applicability across heterogeneous segmentation scenarios.

### Visual performance evaluation

Figure 7 presents qualitative comparisons highlighting the visual performance of Dense121GAN against UNet and ResNet-based GANs across representative samples from multiple datasets. Columns correspond to (a) the example input image, (b) ground-truth mask, (c) Dense121GAN prediction, (d) Dense121GAN confusion map, (e) ResNet prediction, (f) ResNet confusion map, (g) UNet prediction, and (h) UNet confusion map. In the confusion maps, True Positives (TP) are shown in white, True Negatives (TN) in black, False Positives (FP) in blue, and False Negatives (FN) in red. These visualizations clarify model behavior and reveal segmentation accuracy relative to ground truth, supporting detailed assessment of performance.

**Figure 7:**
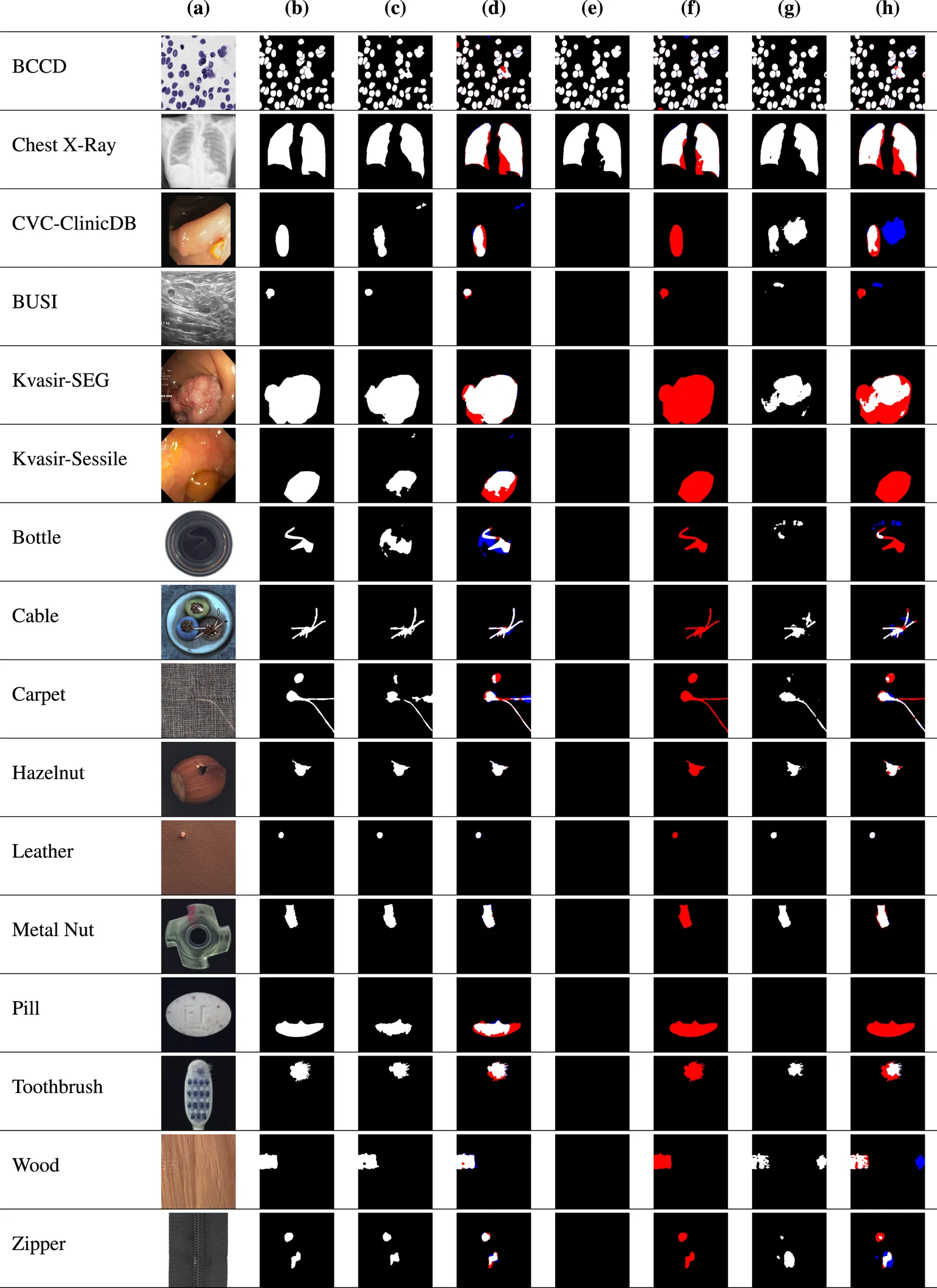
Qualitative comparison of segmentation outputs across the evaluated medical and industrial datasets. Columns denote: (A) example input image, (B) ground-truth mask of the example image, (C) Dense121GAN prediction, (D) Dense121GAN confusion map, (E) ResNet prediction, (F) ResNet confusion map, (G) UNet prediction, and (H) UNet confusion map. In the confusion maps, white indicates true positives, black indicates true negatives, red indicates false negatives, and blue indicates false positives.

Beyond overall visual quality, dataset-specific results further emphasize the strengths of Dense121GAN over the baseline models. In the Cable dataset, Dense121GAN captures fine insulation cuts and wire misalignments, while UNet and ResNet miss or mislocalize them, producing FN-dominated confusion maps. For Kvasir-Sessile, where sessile polyps are flat and low-contrast, Dense121GAN preserves contours effectively, whereas UNet is overly conservative and ResNet collapses. In BUSI, Dense121GAN delineates tumors sharply despite ultrasound noise, while UNet struggles under low contrast conditions and ResNet collapses completely.

For CVC-ClinicDB, Dense121GAN handles glare and motion blur robustly, unlike ResNet’s near-empty outputs and UNet’s less stable segmentation. In Bottle, Dense121GAN isolates neck defects completely, while UNet detects only partial contours and ResNet fails entirely. On Carpet, Dense121GAN aligns masks closely with defect boundaries, whereas ResNet generalizes poorly and UNet produces smoother but less precise predictions.

On Hazelnut, Dense121GAN captures fine cracks and contamination missed by the baseline models. In Leather, it detects cuts and folds with minimal false positives, while UNet introduces additional false regions and ResNet misses defects entirely. On Metal Nut, Dense121GAN accurately segments scratches and dents, outperforming the blurred or collapsed predictions of the baseline models.

For Pill, Dense121GAN identifies small cracks and imprint errors, while UNet and ResNet fail to preserve the defect structures. In Toothbrush, Dense121GAN resolves fine bristle defects more accurately, whereas the baseline models either hallucinate artifacts or underperform. On Wood, Dense121GAN maintains consistent predictions on subtle defects, outperforming both noisy ResNet outputs and less precise UNet predictions. In Zipper, Dense121GAN segments misalignments and fabric defects reliably, unlike inconsistent UNet predictions and non-functional ResNet outputs.

Finally, in the BCCD and Chest X-Ray datasets, even where ResNet achieved similar quantitative scores, Dense121GAN produced more spatially accurate masks, particularly for overlapping blood cells and lung boundaries. These qualitative observations align with the quantitative results presented in Tables 1 and 2, confirming that Dense121GAN maintains strong spatial precision, avoids mode collapse, and effectively segments complex and subtle structures across both medical and industrial domains.

### Ablation study

To further analyze the architectural design choices of Dense121GAN, an ablation study was conducted on the CVC-ClinicDB dataset using the same 50-epoch training schedule and identical five-fold cross-validation splits adopted throughout this work.

The study evaluates two main factors: encoder freezing and the number of residual skip connections. For encoder freezing, the default frozen DenseNet121 configuration was compared against a partially fine-tuned variant in which the final DenseNet block was unfrozen. For skip connections, configurations with four, two, and zero residual skip connections were evaluated.

Table 6 summarizes the obtained results. The experiments show that the frozen encoder configuration consistently outperformed partial fine-tuning under the fixed training setting adopted in this work. In addition, reducing the number of residual skip connections resulted in noticeable performance degradation across all evaluation metrics, confirming the importance of multi-scale spatial feature preservation during reconstruction. The strongest performance was achieved using the frozen encoder with four residual skip connections, which was therefore adopted as the default Dense121GAN configuration throughout the study.

**Table 6:** Ablation study of Dense121GAN on CVC-ClinicDB. All experiments were conducted for 50 epochs using identical five-fold cross-validation splits. *F* denotes frozen encoder, *U* denotes partial fine-tuning (last DenseNet block unfrozen), and ${S_{k}}$ denotes the number of skip connections.

Variant	IoU	Dice	MCC	Kappa	SSIM
Dense121GAN (F + ${S_{4}}$)	0.8411	0.9034	0.8996	0.8956	0.9604
Dense121GAN (F + ${S_{2}}$)	0.6923	0.8180	0.8025	0.7980	0.9240
Dense121GAN (F + ${S_{0}}$)	0.5714	0.7285	0.6950	0.6895	0.8910
Dense121GAN (U + ${S_{4}}$)	0.6185	0.7640	0.7320	0.7265	0.9020
Dense121GAN (U + ${S_{2}}$)	0.5620	0.7160	0.6850	0.6790	0.8880
Dense121GAN (U + ${S_{0}}$)	0.4720	0.6420	0.6020	0.5960	0.8610

These findings support the adopted Dense121GAN design choices and further demonstrate the contribution of residual skip connections and pretrained feature preservation to segmentation stability and reconstruction quality.

## Discussion

### Analysis of classification metrics and robustness to class imbalance

To provide a more comprehensive evaluation of segmentation performance, additional classification metrics, including Matthews Correlation Coefficient (MCC) and Cohen’s Kappa, were analyzed alongside overlap-based metrics such as IoU and Dice. In general, models achieving higher IoU and Dice scores also exhibited higher MCC and Kappa values, indicating consistent agreement between predicted and ground truth masks across both foreground and background regions. This trend confirms that Dense121GAN improves not only overlap-based segmentation accuracy but also class-balanced prediction quality across heterogeneous datasets.

A notable exception was observed in several datasets where the ResNet-based generator exhibited complete failure, resulting in IoU and Dice scores of 0.0000 (Table 1). Examination of the predicted masks and confusion maps (Fig. 7) confirmed that these failures correspond to severe mode collapse behavior, where the generator produced near-empty or predominantly background segmentation masks. Consequently, extremely low or near-zero IoU and Dice scores were observed in some datasets. This behavior was particularly evident in challenging scenarios characterized by severe class imbalance, limited training samples, subtle object boundaries, or high structural variability, which increased optimization instability during adversarial training. Importantly, the same ResNet-based generator achieved competitive performance on datasets such as BCCD and Chest X-Ray, indicating that the observed failures were not caused by implementation or evaluation errors. Instead, they were strongly associated with dataset characteristics and adversarial training dynamics.

This behavior was particularly evident in datasets containing small, low-contrast, or structurally irregular targets. In CVC-ClinicDB, Kvasir-SEG, and especially Kvasir-Sessile, the target polyps are often small, flat, and visually similar to surrounding tissue. BUSI images introduce additional difficulty due to speckle noise and weak lesion boundaries. Similarly, several MVTec categories, including Cable, Pill, Toothbrush, Wood, Zipper, Bottle, and Metal Nut, contain thin, fragmented, or spatially limited defects occupying only a small portion of the image. These conditions create severe foreground-background imbalance and increase the difficulty of adversarial optimization.

Under such conditions, the ResNet-based generator frequently collapsed toward trivial background-dominated predictions. This behavior is visible in the confusion maps, where large false-negative regions and almost no true-positive detections are observed in datasets such as Kvasir-Sessile and Cable. In contrast, Dense121GAN preserved substantially more foreground structure and produced spatially coherent predictions under identical training conditions.

One possible explanation is related to the architectural differences between the generators. Unlike Dense121GAN and UNet, the ResNet-based generator does not employ encoder-decoder skip connections that directly propagate high-resolution spatial information to the reconstruction stages. While this design is often effective for classification-oriented feature abstraction, it can become problematic in image generation and segmentation tasks requiring preservation of fine structural details.

In datasets containing small or subtle targets, repeated downsampling and residual transformations may suppress weak foreground cues, increasing the likelihood of adversarial collapse toward background predictions. In contrast, the residual skip connections used in Dense121GAN preserve local structures, boundary information, and fine-grained spatial features throughout reconstruction, improving both stability and segmentation fidelity.

This interpretation is further supported by the dataset-dependent behavior of the ResNet-based generator. In datasets such as BCCD and Chest X-Ray, foreground regions occupy a larger portion of the image and exhibit more stable visual characteristics. Under these conditions, the ResNet-based generator remained stable and achieved competitive performance. However, as target size decreased and structural complexity increased, the generator became increasingly vulnerable to collapse.

The results also highlight the importance of balanced evaluation metrics in highly imbalanced segmentation tasks. Despite producing degenerate outputs, the collapsed ResNet-based generator often achieved artificially high specificity and negative predictive value (NPV), since most pixels belong to the background class. Consequently, background-dominated predictions can yield high true-negative rates while completely failing to segment the target regions. Metrics such as IoU, Dice, MCC, and Cohen’s Kappa more reliably expose these failures because they penalize the absence of true-positive predictions and incorporate multiple components of the confusion matrix.

In particular, MCC and Cohen’s Kappa proved especially informative in detecting collapse behavior. For degenerate predictions, both metrics dropped close to zero, indicating minimal agreement with the ground truth despite high background accuracy. Across both medical and industrial datasets, Dense121GAN consistently achieved higher MCC and Kappa values than UNet and ResNet-based generators. This demonstrates that the proposed architecture improves not only segmentation accuracy but also robustness against imbalance-induced bias, unstable adversarial optimization, and mode collapse.

Overall, the inclusion of MCC and Cohen’s Kappa provides a more reliable and interpretable evaluation framework for segmentation tasks involving small or sparse foreground regions. The results further demonstrate that Dense121GAN maintains stable and balanced predictions across diverse datasets where conventional adversarial generators may fail.

## Conclusion

This study introduced Dense121GAN, a conditional generative adversarial network that combines a frozen DenseNet121 encoder with residual skip connections for robust image segmentation. The proposed framework leverages transfer learning to improve feature extraction while preserving gradient flow and spatial fidelity through residual pathways. Extensive experiments conducted across 16 diverse medical and industrial datasets under a unified evaluation framework demonstrated that Dense121GAN generally outperformed baseline UNet and ResNet-based GANs in overlap-based segmentation accuracy, balanced classification metrics, regression-based reconstruction quality, convergence speed, and training stability. The model showed particularly strong performance in challenging scenarios involving small objects, subtle defects, complex textures, and high structural variability, supporting its robustness across heterogeneous segmentation tasks. In addition, Dense121GAN maintained competitive performance even on relatively small datasets, highlighting its suitability for domains with limited annotated data.

Despite these promising results, several limitations should be acknowledged. All experiments were conducted using images resized to a fixed resolution of 256×256 pixels to ensure stable training and fair comparison, which may reduce fine-grained details available in higher-resolution images. Furthermore, the DenseNet121 encoder remained frozen during training to reduce computational cost and mitigate overfitting on limited datasets; however, this design may restrict adaptation to domain-specific image characteristics. Data augmentation was also applied only to industrial datasets, while medical datasets remained unaugmented to preserve pathological realism, potentially introducing differences in robustness and generalization behavior across domains.

Additionally, the unified cross-validation protocol adopted in this study improves consistency across heterogeneous datasets but may limit direct comparability with prior works that use official dataset-specific splits and evaluation procedures. Differences in annotation strategies, task definitions, and dataset structures may also introduce dataset-dependent biases. Moreover, exhaustively evaluating all possible experimental configurations, including alternative image resolutions, encoder fine-tuning strategies, augmentation schemes, and architectural variants, remains computationally challenging.

Future work will investigate these factors in greater detail to better understand their individual and combined effects on segmentation performance. Additional extensions will explore integrating attention mechanisms into the generator to improve context-aware feature modeling and localization of subtle regions. Lightweight variants of Dense121GAN will also be investigated for deployment on resource-constrained devices and real-time clinical or industrial applications.

Another important direction involves extending Dense121GAN to multi-class segmentation tasks. This can be achieved by modifying the generator output to produce multi-channel segmentation maps and by adopting suitable multi-class loss functions, such as categorical cross-entropy or generalized Dice loss. The discriminator may also be adapted to evaluate consistency between input images and multi-class segmentation outputs.

However, multi-class segmentation introduces additional challenges, including increased class imbalance, higher inter-class ambiguity, and more complex spatial relationships between structures. These factors may negatively affect adversarial training stability and require additional regularization strategies or class-aware loss weighting.

Despite these challenges, the dense feature reuse, stable optimization behavior, and robust adversarial learning demonstrated by Dense121GAN indicate strong potential for future multi-class and volumetric segmentation applications. Future studies will therefore investigate these extensions across more complex medical and industrial imaging scenarios, including 3D volumetric segmentation tasks.

## Additional Information and Declarations

### Data Availability

The following information was supplied regarding data availability:

The data and code are available at Zenodo: Davud, M. (2025). Dense121GAN: cGAN Framework for Medical and Industrial Image Segmentation (Version 1). Zenodo. https://doi.org/10.5281/zenodo.17935252.
